# Genomic Instability in the Lymphocytes of Dogs with Squamous Cell Carcinoma

**DOI:** 10.3390/ani14192754

**Published:** 2024-09-24

**Authors:** Ewa Wójcik, Emilia Kot, Iga Wójcik, Anna Wysokińska, Paulius Matusevičius

**Affiliations:** 1 Institute of Animal Science and Fisheries, University of Siedlce, 08-110 Siedlce, Poland; emiliakot63@gmail.com (E.K.); igawojcik03@gmail.com (I.W.); anna.wysokinska@uws.edu.pl (A.W.); 2 Department of Animal Nutrition, Lithuanian University of Health Sciences, LT-47181 Kaunas, Lithuania; paulius.matusevicius@lsmu.lt

**Keywords:** dog, cancer, genome instability, sister chromatid exchange assay, alkaline comet assay

## Abstract

**Simple Summary:**

The aim of the study was to identify chromosome damage using the sister chromatid exchange assay and DNA fragmentation by the comet assay in dogs with cancer, as well as to determine the suitability of these techniques for assessing chromatin stability in healthy and sick dogs (with squamous cell carcinoma). The genetic assays are very sensitive and can be used as biomarkers of normal DNA replication and repair potential and the maintenance of control over the entire cell cycle. The use of the cytogenetic tests will enable a more precise assessment of genome stability and integrity in animals and make it possible to determine the number of chromosomal instabilities generated in a given individual, which can be indicative of its health status.

**Abstract:**

Genome instability is a characteristic trait of tumours and includes changes in DNA and in chromosomes. The aim of the study was to identify chromosome damage using the sister chromatid exchange assay and DNA fragmentation by the comet assay in dogs with cancer, as well as to determine the suitability of these techniques for the assessment of chromatin stability in healthy and sick dogs. The assays identified genomic instabilities in dogs with cancer (squamous cell carcinoma) and in healthy dogs. The genetic assays are very sensitive and can be used as biomarkers of normal DNA replication and repair potential and the maintenance of control over the entire cell cycle. The use of the cytogenetic tests will enable the more precise assessment of genome stability and integrity in animals and make it possible to determine the number of chromosomal instabilities generated in a given individual, which can be indicative of its health status. The identification of instabilities can be used in routine diagnostic examination in dogs with cancer for more accurate diagnosis and prognosis.

## 1. Introduction

During the neoplastic process, cells with damaged genetic material grow in an uncontrolled manner. These cells have unlimited proliferative potential, are self-sufficient in terms of growth factors, and are not susceptible to either apoptotic or antiproliferative factors. Tumour formation requires a number of successive mutations, as well as individual predispositions, which can be heritable. Certain traits, such as overweight, obesity, or reduced immunity, make dogs more susceptible to cancer. Tumours induce a number of changes in the body, e.g., inflammation, immune impairment, and nutrient depletion [1]. The neoplastic process is closely linked to the ageing processes of the cell and body. Cancer is the cause of death in 8 out of 1000 adult dogs and in as many as 50% at the age of eight years [2,3]. Squamous cell carcinoma is one of the most common cancers of the skin, head and neck in dogs [4]. It often occurs on skin with little or no hair and little pigmentation, which is exposed to exogenous factors such as UV radiation [5]. Squamous cell carcinoma (SCC), sometimes known as spinocellular carcinoma, is most common on the skin and mucous membranes. It may be keratinizing, with keratinization penetrating deep into the skin or only on the tumour surface. Another form is squamous papilloma, which is generally benign. SCC is most often an irregular proliferative or ulcerated lesion–infiltrative, brittle, haemorrhagic, and painful. It can be treated surgically, or with radiation therapy in the case of inoperable tumours [6,7]. Published research on SCC in dogs has not conclusively identified its cause [8,9,10]. A genome-wide association study conducted by Karyadi et al. [9] showed that SCC occurs more often in dark-haired dogs.

According to Liu et al. [7], canine cancers reflect numerous molecular features of human SCC at various levels (e.g., anomalies in the number of genome copies, mutations in sequences, repeating changes in genes and SCC pathways, and molecular heterogeneity). According to the authors, studies on spontaneous canine SCC can be a very useful model in preclinical studies of human disease. As companion animals, dogs live in the same environmental conditions as humans, with varying exposure to carcinogenic agents and risk of diseases to civilization, but they suffer from cancer more often than humans [11,12]. As the aetiopathogenesis, morphology and biological behaviour of tumours in dogs and humans are similar, comparative studies on mutations and the formation of a pathological phenotype in dogs can be crucial models in the diagnosis of human diseases [13,14,15]. In human cancer cells, many changes in genetic material are observed. The occurrence of instabilities is influenced by many mutagenic and carcinogenic agents, both exogenous and endogenous, which induce errors in the replication, transcription and translation processes and abnormal functioning of DNA repair mechanisms and cell cycle checkpoints. Unrepaired and poorly repaired DNA damage adversely affects genome integrity. Abnormalities that arise during somatic cell division and especially during mechanisms maintaining genome stability lead to disturbances in the body’s development and health [16,17]. The p53 gene is an important tumour suppressor gene involved in DNA repair, proliferation, and apoptosis. For this reason, it is referred to as the ‘guardian of the genome’. A mutation of this gene entails the loss of its function, the formation of chromosomal instabilities, and the risk of cancer in dogs and humans [13]. Latent chromosomal instability is expressed as the accumulation of various changes in chromosome number structure. It is detected in vitro following the addition of compounds that induce chromosome damage to a cell culture. The onset and development of cancer as the result of genome instability are therefore highly complex and multi-faceted processes. According to Tanaka and Hirota [18], instability is closely linked to cancer cells. Cytogenetics deals with the analysis of the constitutional karyotype and with chromosomal abnormalities, e.g., the detection of chromosomal aberrations associated with the tumour and other types of instabilities. Dogs have a diploid chromosome number of 78. Only sex chromosomes have two arms, while autosomal chromosomes are acrocentric. For this reason, this specific karyotype poses many difficulties in cytogenetic analyses in dogs. Reimann-Berg et al. [14] reported a high degree of homology of human and canine chromosomes. Due to this high similarity, cytogenetic tests used in humans can be used in dogs (and vice versa). Damage identified using cytogenetic tests can be used as diagnostic and prognostic markers in studies on squamous cell carcinoma in dogs. Cytogenetic assays that can be used to identify chromosomal instabilities include the sister chromatid exchange assay (SCE) and alkaline comet assay (SCGE). Cytogenetic tests make it possible to identify various types of damage to genetic material, errors occurring during replication and transcription, and DNA strand breaks. When these errors are not prepared or improperly repaired, they negatively affect the stability of the genome. Unstable sites in the genome are highly susceptible to the deregulation of replication and transcription and can initiate the neoplastic process [19,20].

Tumour formation depends on many epigenetic and genetic factors, which can be active or dormant, but also on the animal’s living environment. According to McGranahan et al. [20], genome instability is a feature of most tumours in humans and of intratumour heterogeneity, unfortunately associated with a poor prognosis and drug resistance. Cytogenetics in dogs has developed considerably in recent years, due to interest in the dog as a biomedical model of genetically determined diseases and to the availability of genome resources. Nevertheless, there are still few studies on the identification of various kinds of lesions in dogs. The occurrence of damage to genetic material in Bernese mountain dogs and St. Bernard dogs can be associated with a predisposition to SCC. Therefore, in the present study, we analysed the frequency of chromosomal instabilities in dogs with cancer. Damage to genetic material in individuals with cancer can be detected in cells isolated directly from a sample of cancer tissue cells, but the patient’s peripheral blood lymphocytes are also an excellent material for this purpose, and they are much easier to collect and process. Another advantage is that they can be cultured quickly in vitro. A standard in vitro lymphocyte culture lasts 72 h, whereas cultures of cells collected from cancerous tumours can take from 10 days to several weeks. Lymphocytes are ideal cells for assessing genetic stability in an animal because as they circulate throughout the body, they are exposed to all possible risk conditions and endogenous mutagenic factors. Hence, the migration of cancer cells through blood vessels together with the blood enables their identification in lymphocyte cultures. Therefore, lymphocytes are an early marker that can easily be analysed, both after their isolation and after in vitro culture. Due to their defensive function, they are the first to receive signals of any negative effects of various factors on genetic material, which generate damage that accumulates in cancer cells. According to Jefford and Irminger-Finger [21], cancers are diagnosed on the basis of clinical examination, systemic factors, and cytogenetic and molecular analyses. At the systemic level, changes in leukocyte levels are monitored, and circulating factors may be associated with tumours. In vitro cultures of these cells aimed at obtaining and analysing chromosomes are good biomarkers. Xun et al. [22] also used peripheral blood lymphocytes to diagnose squamous cell carcinoma of the larynx, regarding them as reliable biomarkers. In addition, Rossner et al. [23] tested the frequency of chromosomal instability in peripheral blood lymphocytes in non-melanoma skin cancer to confirm the hypothesis of a positive correlation between the frequency of instability in lymphocytes and cancer risk. Ribeiro et al. [24] and Han et al. [25] also found that lymphocytes are suitable cells for the identification of genomic instability in squamous cell carcinoma. These effects can be visualized by analysing these cells with the use of various techniques, such as the alkaline comet assay, which reveals global damage in these cells, as well as techniques using mitotic chromosomes obtained in in vitro cultures and assessed using tests such as the SCE assay. The comet assay performed on lymphocytes has been used in studies by Ribeiro et al. 2008, Sztyfter [26] and Kamlesh et al. [27] to identify genomic instability in patients with cervical cancer, while the SCE assay has been used to detect chromosomal instability in patients with cervical cancer [28]. These researchers noted that the frequency of SCEs in the peripheral blood lymphocytes of the patients was significantly higher than in healthy individuals. Salawu et al. [29] identified SCEs in patients with soft tissue sarcoma. The authors identified instabilities in lymphocytes. Similarly, Vodicka et al. [30] analysed chromosome damage in the peripheral blood lymphocytes of cancer patients (with various malignant tumours) and found a much higher frequency of genomic instability in cancer patients than in healthy subjects. Thus, the identification of genomic instability in the lymphocytes of dogs suffering from squamous cell carcinoma is clearly a suitable method for determining its level in these individuals.

The aim of the study was to identify genomic instability in lymphocytes of dogs with squamous cell carcinoma using SCE and comet assays.

## 2. Materials and Methods

### 2.1. Ethics Statement

The study was carried out in strict compliance with Directive 63/2010/EU and the *Journal of Laws* of the Republic of Poland of 2015 on the protection of animals used for scientific or educational purposes. The tests performed were non-invasive, which means that, in accordance with the Directive, they did not cause pain, suffering, distress, or permanent damage to an extent equal to, or more severe than, a needle-stick injury. Blood from dogs was obtained during veterinary examinations. The study was approved by the Polish Local Ethics Committee, Warsaw, Poland (Number: 51/2015) and by the Polish Laboratory Animal Science Association (Numbers 3235/2015; 4466/2017). The study was carried out in accordance with ARRIVE guidelines.

### 2.2. Animals

The material for the study was peripheral blood drawn from the basilic vein of Molosser-type dogs of two breeds (St. Bernard dogs (B1) and Bernese mountain dogs (B2)), 20 cases and 10 controls per breed, with an equal number of males (M) and females (F) among cases and controls (healthy (H), cancer (C)). Blood was drawn during routine veterinary activities from dogs in which cancerous changes had been diagnosed by a veterinarian. The age of the animals ranged from 8 to 12 years.

### 2.3. Cell Culture

A cell culture of peripheral blood lymphocytes was grown on Lymphogrow medium (CytoGen, Greven, Germany). Lymphocytes were cultured in vitro (culture time 72 h, temperature 37.5 °C, 5% CO_2_, with stable humidity) and then treated with a hypotonic solution and fixed. Cytogenetic analyses to identify damage to genetic material were performed using the SCE and comet assays. Colchicine was added (2.5 µg/mL^−1^) 3 h before the termination of the culture. For the culture for the SCE assay, BrdU (5-bromodeoxyuridine) was added (10 µg/mL^−1^) after 24 h of the cell culture. After 72 h, a hypotonic solution (0.56% KCl, temperature 37 °C) was added to the suspension, and then it was fixed with Carnoy’s solution (3:1 methanol-acetic acid).

### 2.4. SCE Assay

Sister chromatid exchanges are the result of the incorrect replication and repair of damage in the G2 phase of the cell cycle. At the site of errors, exchanges take place between chromatids of the same chromosome. SCE is coupled with recombination repair, gene amplification, induction of point mutations, and cytotoxicity [19]. The SCE assay is a very sensitive and reliable biomarker used to detect changes in chromosomes at an early stage. This is a natural process taking place in every cell cycle, but it can be induced by exposure to mutagenic and carcinogenic agents [12]. Both HR (homologous recombination) and G2 checkpoint genes are necessary mechanisms essential for the identification of double-strand breaks (DSB).

Microscope slides were prepared according to a modified FPG (fluorescence plus Giemsa) procedure [31]. The staining procedure involved digestion with 0.01% RNase (1 h, 37 °C), incubation in a solution of 0.5 × SSC with Hoechst 33258 (1 h, room temperature), incubation in 0.5× SSC with simultaneous UV irradiation (17.28 mJ/cm^2^) (0.5 h, room temperature), overnight incubation at 4 °C in a solution of 0.5× SSC, UV irradiation (17.28 mJ/cm^2^) (0.5 h, room temperature), incubation in a solution of 0.5× SSC (2 h, 58 °C), and 4% Giemsa staining (1 h, room temperature). The number of SCEs in the cells of each animal was counted. Twenty metaphases from each individual were examined. 

### 2.5. SCGE Assay

The alkaline comet test does not require an in vitro cell culture. It can be used to analyse isolated single cell nuclei immobilized in an agarose gel on microscope slides, which are subjected to lysis, so that DNA is released from the cell nucleus [32,33]. This assay identifies global DNA damage [23].

The SCGE (single cell gel electrophoresis) technique according to Singh et al. [34] was used to identify DNA damage. Lymphocytes were isolated from the blood using Histopaque—1077. Isolated lymphocytes mixed with 0.5% LMP (low melting point) agarose gel were spotted on microscope slides covered with 0.5% NMP (normal melting point) agarose and incubated at 4 °C. Then the lymphocytes were embedded in a 0.5% LMP agarose gel. The specimens were subjected to alkaline lysis (2.5 M NaCl, 100 mM Na2EDTA, 0.4 M Tris-HCl, 1% sodium N-lauroylsarcosinate, 10% Triton X-100, 1% DMSO, pH = 10) (overnight, 4 °C), relaxation (0.5 h), electrophoresis (25 V, 300 mA, 20 min, without access to light, 20 min), neutralization with Tris-HCl (3 × 5 min, room temperature), drying at 38 °C, and staining with ethidium bromide (overnight, 4 °C). DNA integrity was determined on the basis of the percentage content of DNA in the tail (%T DNA) of the comet. Fifty cells were analysed for each animal. Changes observed in cells were classified according to Gedik’s scale: N—no DNA damage or less than 5% damage in the comet tail; L—low level of damage (5–25%); M—moderate damage (25–40%); H—high level of damage (40–95%) and T—over 95% DNA damage [35].

### 2.6. Analysis

An Olympus BX53 microscope was used for microscopic analysis. MultiScan image analysis software v.14.02 from Computer Scanning Systems was used to analyse damage to chromosomes stained using the SCE assay. CASP 1.2.2 software was used to analyse the degraded DNA of dog lymphocytes identified by the comet assay [36]. The results were subjected to statistical analysis using Statistica 13.1 PL software. Means between individuals within groups were compared by one-way analysis of variance. The assays were analysed by three-way analysis of variance. The significance of differences between means for a given type of instability within factors was assessed by Tukey’s test (*p* < 0.05).

## 3. Results

**Figure 1:**
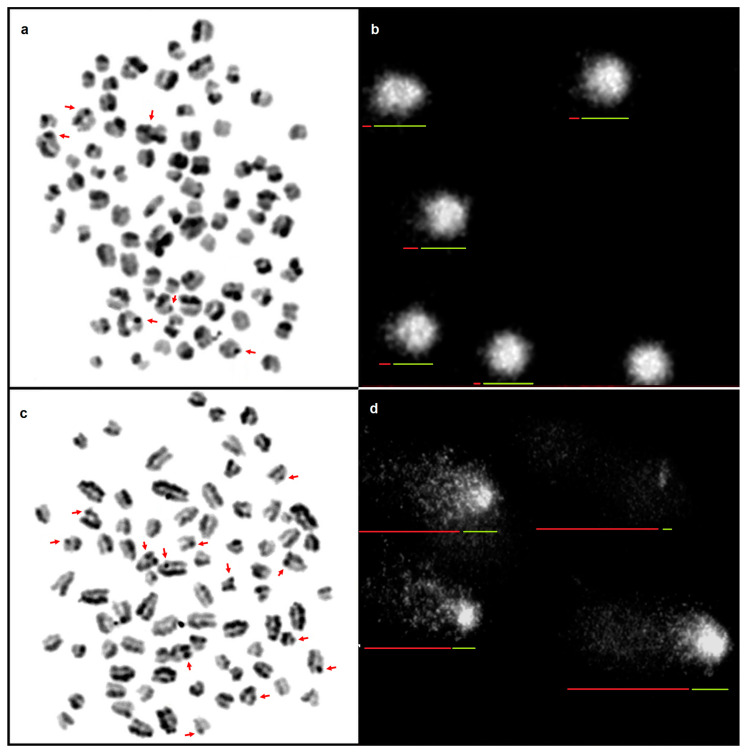
Mitotic chromosomes in the metaphase of a healthy dog stained by the (**a**) SCE assay; SCEs indicated with arrows; and (**b**) cell nuclei of lymphocytes subjected to the SCGE assay; arrows indicate the tail (red) and head (green) of the comet. Mitotic chromosomes in the metaphase of a sick dog stained by the (**c**) SCE assay; SCEs indicated with arrows; and (**d**) cell nuclei of lymphocytes subjected to the SCGE assay; arrows indicate the tail (red) and head (green) of the comet. Scale bar 10 µm.

Chromatin instabilities were analysed in two breeds of Molosser-type dogs with cancer and in clinically healthy dogs (Figure 1).

### 3.1. SCE Assay

The average frequency of SCEs for healthy dogs was one-third of the frequency in sick dogs. Statistically significant differences were noted between healthy and sick dogs. The average frequency of SCEs for males and females was similar, and the minor differences observed were not statistically significant. Table 1 presents the statistical characteristics of the analysed chromosome instabilities in the dogs. In healthy dogs, for breed 1, the average frequency of SCEs/cell was one third of the frequency in sick dogs. The differences in SCE frequency were statistically significant. The average frequency of SCEs within groups for males and females was similar. Similarly, the average SCE frequency between healthy and sick dogs was similar. Differences were noted between sick and healthy dogs; in the case of both males and females, SCEs/cell were three times as high in sick dogs as in healthy dogs. For B2, the average frequencies of SCEs had a similar distribution of values as in B1 (Table 2). The differences in the frequency of SCE between healthy and sick dogs within breeds were statistically significant, while there were no statistically significant differences between males and females. Differences were observed in the frequency of SCEs between individual dogs of both breeds within the groups of healthy and sick dogs (Supplementary Material Table S1).

### 3.2. SCGE Assay

The %T DNA value obtained for sick dogs was in many cases as high as in healthy dogs. The differences observed in the amount of fragmented DNA between healthy and sick dogs were statistically significant. Statistically significant differences were also found in the amount of fragmented DNA for healthy and sick dogs within breeds 1 and 2 (B1: 1.7 and 34.8; B2: 3.2 and 35.3) and within the same sex in healthy and sick dogs. No statistically significant differences were found between sexes in either breed in the amount of damage identified by the SCGE assay. A similar pattern was observed for sex in the group of healthy and sick dogs. Table 1 and Table 2 present DNA damage identified by the comet test in the chromosomes of the groups of dogs. Differences were observed in the frequency of DNA fragmentation (%T DNA) between individual dogs in both breed B1 and B2 within the groups of healthy and sick dogs (Supplementary Material Table S1). An additional criterion, Gedik’s scale, was used to assess the DNA integrity of the dogs. Two levels of damage were noted in the healthy dogs: N in 16 dogs and L in 4 dogs. Levels N and L are associated with a low level of DNA damage. The levels of damage in the other group were M (16 dogs) and H (4 dogs).

## 4. Discussion

Recent years have seen interest in cytogenetic testing to study the effect of environmental and genetic factors and living conditions on the integrity of genetic material in animals. This is important because many diseases are the result of mutations and genome instabilities. One of the diseases resulting from genome instability is cancer, which arises from unlimited and uncontrolled cell division. In the normal cell cycle, dead cells are replaced with new ones with normal structure. Sometimes, however, cells with altered genetic material appear and do not perform their function. Oncogenesis usually begins with a single mutation or one of the forms of instability in the genetic material of the cell. As a result of poorly functioning checkpoints and repair systems, the error goes unnoticed or is incorrectly repaired, which leads to the formation of a tumour [2,7,13,37,38]. Squamous cell tumours affecting several epithelial cell lines (of both the epidermis and mucous membranes) are among the most common tumours in dogs [4,5]. They are usually located on the muzzle, trunk, abdomen, limbs, scrotum, lips, nail bed, or other sites on the skin [39,40,41]. Excessive exposure to sunlight and environmental pollution in urban and industrial areas are considered to be carcinogenic factors. Unfavourable exogenous factors can be introduced during breathing or feeding. Jasik and Reichert [11] and Nowak and Madej [41], in analyses of tumours occurring in dogs in Poland, found that as many as 44–50.3% of them were epithelial tumours, including 66% malignant tumours. The diagnosis of tumours in dogs is a long process. Cytogenetic and molecular tests require time, during which the tumours can continue to develop spontaneously. Chromosomal instabilities identified by scientists who have studied dogs with epithelial tumours, both benign and malignant, include centric fusions, structural chromosomal aberrations, and numerical chromosomal aberrations, most commonly aneuploidy [42]. The identification of the type of mutation influences the further treatment undertaken [43]. There has been little interest in the identification of other chromosomal instabilities in dogs, identified using the SCE and SCGE assays. Changes taking place in genetic material accelerate ageing in cells and lead to the development of many diseases. The SCE assay has been used to identify instabilities in research on tumours in humans. The researchers noted a higher frequency of SCEs in cancer patients [44,45,46]. Unfortunately, there is no information on this type of instability in healthy dogs or dogs with cancer. The SCE assay used to identify chromosomal instabilities is a pioneering test. In the present study, the SCE assay revealed that the level of damage in sick dogs was three times that noted in healthy dogs. The analyses in the present study identifying chromosomal instabilities provide new scientific information about the genome of these animals. According to Wilson and Thompson [19], the number of spontaneous SCEs in a normal cell cycle is low. In the present study, the average frequency of SCEs in healthy dogs was 5.2. SCE frequency increases in animals in the case of disease [17,47]. In the present study, we also found that the frequency of SCEs in dogs with cancer was three times as high as in their healthy counterparts. High frequencies of SCEs in the lymphocytes of patients with various cancers have been reported by Dhillon et al. [28], Salawu et al. [29], and Cortés-Gutiérrez et al. [48]. In some disease syndromes in humans, very high SCE frequencies are observed as a result of mutation of genes responsible for maintaining stability. Often a fivefold increase in HR is observed during DSB repair, while in the case of damage to the ‘genome guardian’ p53, the rate of HR increases more than tenfold [49].

The comet assay is the cytogenetic test most often used in people and animals. It can be used to examine many individual cell nuclei at the same time, identifying a wide variety of DNA lesions owing to a multi-stage procedure. The SCGE method enables the quantification of the scale of DNA damage in vitro [32,33]. The comet assay is one of the most sensitive methods for identifying oxidative damage in cancer. It is fast and simple, and observations at the level of a single cell are free of artefacts [32]. In healthy people, the level of DNA damage in the study cited was low—up to 6 (%T DNA). In the case of diseased cells, the average %T DNA increased to 24 in the case of colorectal cancer and 12 in the case of breast cancer. In the present study, low values were obtained for healthy dogs (2.4 (%T DNA)) and high values for sick dogs (43.9). There is little information on the use of the comet assay in dogs [32]. Ribeiro et al. [24] analysed the frequency of genomic instability using the comet assay on lymphocytes in rats with oral squamous cell carcinoma. Their results showed an increased frequency of DNA damage in the blood cells. Kamlesh et al. [27] used the same test to study the frequency of genomic instability in oral squamous cell carcinoma, regarding it as a highly reliable biomarker. Heaton et al. [50] used the comet assay to identify oxidative damage in the leukocytes of healthy dogs. Pereira et al. [51] tested DNA integrity in the sperm of dogs, using the comet test to determine the level of oxidative damage. Perez et al. [52] also used the comet test to examine DNA damage induced by cigarette smoke in dogs. In the present study, low %T DNA was also obtained in healthy dogs, indicating high chromosomal stability in these animals. The %T DNA in sick dogs was very high in comparison to healthy dogs (43.9 and 0.1, respectively).

A very important factor influencing the level of damage to genetic material is age. Many scientists dealing with the identification of chromosomal instabilities have observed an increase in the level of both DNA and chromosome damage in old animals [53]. During the ageing process, many irreversible changes take place in the genome, which are fixed in cell divisions, resulting in high susceptibility to disease. The long-term exposure of genetic material to mutagenic and carcinogenic agents results in a large number of chromosomal instabilities observed with the passage of time, which decreases genome integrity and increases the rate of tumour formation in older individuals [17]. The higher level of instability in the dogs in the present study may have been due to age, as the material was from older dogs (8–14 years). According to Sapierzyński [38] and Dernell et al. [54], the risk of cancer in dogs increases with age, and the most common age is 9–10 years. These researchers observed many different types of instabilities in the tumours of the animals, and the heterogeneity and resulting variation in the cells contribute to unpredictable changes at the biological level. Similar observations were made by Morais et al. [42], who reported numerous structural and numerical mutations in a 14-year-old poodle with mammary cancer. Advanced age in dogs with cancer is correlated with a poorer prognosis and a shorter survival time [12,55]. Cancer is diagnosed most often in dogs eight years old and older and is the cause of their deaths [2]. Soukup et al. [56] diagnosed papillary squamous cell carcinoma in large dogs over the age of five years. According to Sapierzyński [38] and Dernell et al. [54], there is a correlation between cancer and the size of dogs. Some tumours are more common in large breeds of dogs: St. Bernard, Doberman, Golden Retriever, Boxer, and German Shepherd [54]. In our study, the St. Bernard dogs and Bernese mountain dogs in which tumours were diagnosed and the level of genome instability was tested were large, Molosser-type dogs. According to Sapierzyński [38], tumours occur in older dogs irrespective of their sex. Nowak and Madej [41] also noted very similar rates of tumour occurrence in male and female dogs (49% and 51%, respectively). Song et al. [57] and Miller et al. [58] found no effect of sex on squamous cell carcinoma. Other scientists analysing other species of animals, both healthy and sick, found no statistically significant differences in the frequency of various forms of genome instabilities between males and females [54]. In the present study, tumour occurrence and the various diagnosed forms of damage to genetic material were not found to be associated with the sex of the animals. According to Kalisz et al. [5], Song et al. [57], and Miller et al. [58], squamous cell tumours are very often diagnosed in old animals, irrespective of breed. In contrast, according to Jasik and Reichert [11], the occurrence of this cancer is dependent on breed, as it has been diagnosed more frequently in certain breeds. In the present study, the analysis of genome instabilities revealed no statistically significant differences in the incidence of damage between the two breeds. This was probably because these breeds were separated fairly recently in the breed creation process, in contrast to primitive, indigenous breeds [59].

## 5. Conclusions

Genome instability is a characteristic feature of tumours, which includes changes in DNA and in chromosomes, and the resulting genetic diversity accelerates oncogenesis. Genomic instability can be detected using peripheral blood lymphocytes, as they circulate throughout the body and are equally exposed to all possible endogenous mutagenic and carcinogenic conditions. The migration of cancer cells with circulating blood allows them to be identified in cultures of white blood cells. The monitoring of changes in the genetic material of leukocytes at the systemic level makes it possible to identify tumour cells, and in vitro cultures of these cells subjected to a cytogenetic assay are good biomarkers. Lymphocytes are an easy and reliable marker revealing damage to genetic material, inefficient cellular mechanisms responsible for identifying and repairing damage, and the disturbed coordination of the apoptosis pathway observed in migrating cancer cells. The sister chromatid exchange assay and alkaline comet assay are ideal tools for identifying damage to genetic material. They identify global DNA damage, replication errors, and malfunctioning cellular mechanisms responsible for genomic stability. The SCEs and fragmented DNA observed in the present study were many times higher in sick dogs than in healthy dogs. As genomic instability has a major influence on cancer progression, the use of suitable cytogenetic tests to identify various forms of chromosome and DNA damage contributes valuable information to canine oncology. Changes in chromosomes not only alter gene expression but also affect genome topology and dynamics, which is of key importance in maintaining the stability of genetic information. The cytogenetic SCE and SCGE assays can be used as biomarkers in SCC in dogs because the identification of instability and the determination of its frequency reveal the potential for DNA repair in the cell and maintenance of control over the entire cell cycle, which is disturbed in sick animals. These tests can have practical applications. They can be used following the treatment process to reveal the effectiveness of the body’s fight against carcinogenic factors, which we can easily observe using lymphocytes.

## Figures and Tables

**Table 1:** Number of instabilities identified in dogs. Means designated with different letters are significantly different at *p* < 0.05.

Sex	SCE		SCGE	
	H	C	H	C
	Mean ± SD		%T DNA	
F	5.3 ^a^ ± 1.7	15.8 ^b^ ± 2.5	1.7 ^a^	35.1 ^b^
M	5.1 ^a^ ± 1.5	15.3 ^b^ ± 2.3	3.1 ^a^	35.1 ^b^
Total	5.2 ^a^ ± 1.6	15.6 ^b^ ± 2.4	2.4 ^a^	35.1 ^b^

M—Males; F—Females; H—Healthy; C—Cancer; %T DNA—percentage content of DNA in the tail of the comet.

**Table 2:** Number of instabilities identified in each breed. Means designated with different letters are significantly different at *p* < 0.05.

Breed	Health	SCE		SCGE	
		F	M	F	M
		Mean ± SD		%T DNA	
B1	H	5.4 ^a^ ± 1.6	5.1 ^a^ ± 1.5	1.7 ^a^	1.7 ^a^
	C	15.7 ^b^ ± 2.5	15.4 ^b^ ± 2.5	33.8 ^b^	35.9 ^b^
B2	H	5.1 ^a^ ± 1.7	5.0 ^a^ ± 1.5	1.8 ^a^	4.6 ^a^
	C	16.0 ^b^ ± 2.6	15.2 ^b^ ± 2.5	36.3 ^b^	34.4 ^b^
Total		10.5 ± 5.7	10.2 ± 5.5	18.4	19.2

B1—St. Bernard dogs; B2—Bernese mountain dogs; M—Males; F—Females; H—Healthy; C—Cancer; %T DNA—percentage content of DNA in the tail of the comet.

## Data Availability

All relevant data are included in the article.

## Supplementary Materials

The following supporting information can be downloaded at: https://www.mdpi.com/article/10.3390/ani14192754/s1, Table S1. Number of instabilities identified in each dog. Means designated with different letters are significantly different at *p* < 0.05.
