# Supplementary material for: Genomic Instability in the Lymphocytes of Dogs with Squamous Cell Carcinoma
Source: Animals (Basel). 2024 Sep 24;14(19):2754. doi: 10.3390/ani14192754 (PMC11476004; doi:10.3390/ani14192754)
Supplement: Supplementary file 1 [file animals-14-02754-s001.zip › animals-3050057-supplementary.pdf]

Table S1. Number of instabilities identified in each dog. Means designated with different letters are significantly different at $p < 0.05$.

Sex	Breed	SCE		SCGE	
		H	C	H	C
		Mean±SD		%T DNA	
		$4.9^{ab}\pm1.6$	$15.5^{b}\pm2.0$	5.1^{b}	43.7^{b}
		$5.5^{b}\pm1.3$	$15.7^{b}\pm2.4$	1.7^{a}	43.9^{b}
	B1	$5.9^{c}\pm1.6$	$16.1^{b}\pm2.6$	0.1^{a}	36.4^{b}
		$5.4^{b}\pm1.6$	$15.1^{ab}\pm3.0$	1.4^{a}	27.5^{a}
		$5.7^{bc}\pm1.6$	$16.0^{b}\pm2.8$	0.1^{a}	28.0^{a}
**F**		$5.5^{b}\pm1.9$	$15.8^{b}\pm2.9$	1.3^{a}	30.0^{a}
		$5.6^{bc}\pm1.9$	$16.2^{b}\pm2.2$	3.1^{b}	32.0^{ab}
	B2	$4.1^{a}\pm1.4$	$15.8^{b}\pm3.0$	4.5^{b}	34.3^{ab}
		$4.8^{ab}\pm1.7$	$15.9^{b}\pm2.4$	0.1^{a}	39.6^{b}
		$5.5^{bc}\pm1.2$	$16.4^{b}\pm2.5$	0.1^{a}	36.0^{b}
		$5.1^{b}\pm1.6$	$15.3^{ab}\pm1.7$	0.2^{a}	37.4^{b}
		$5.5^{b}\pm1.7$	$15.4^{b}\pm2.1$	0.3^{a}	30.4^{a}
	B1	$4.5^{a}\pm1.6$	$14.8^{a}\pm2.0$	4.5^{b}	34.8^{ab}
		$5.3^{b}\pm1.4$	$15.6^{b}\pm1.2$	2.1^{ab}	40.9^{b}
		$5.0^{ab}\pm1.5$	$15.8^{b}\pm2.5$	1.5^{a}	25.3^{a}
**M**		$4.8^{ab}\pm1.6$	$15.1^{b}\pm2.4$	6.2^{b}	28.4^{a}
		$4.5^{a}\pm1.5$	$14.3^{a}\pm2.1$	8.0^{b}	35.9^{b}
	B2	$5.4^{b}\pm1.5$	$15.8^{b}\pm3.0$	8.5^{b}	37.0^{b}
		$5.1^{b}\pm1.3$	$15.2^{b}\pm2.5$	0.1^{a}	41.4^{b}
		$5.5^{b}\pm1.6$	$16.0^{b}\pm2.2$	0.1^{a}	38.9^{b}

M – Males; F – Females; B1 – St. Bernard dogs; B2 – Bernese mountain dogs; H – Healthy; C – Cancer; %T DNA – percentage content of DNA in the tail of the comet
